# Natural Membrane Differentiates Human Adipose-Derived Mesenchymal Stem Cells to Neurospheres by Mechanotransduction Related to YAP and AMOT Proteins

**DOI:** 10.3390/membranes11090687

**Published:** 2021-09-05

**Authors:** Nathalia Barth de Oliveira, Ana Carolina Irioda, Priscila Elias Ferreira Stricker, Bassam Felipe Mogharbel, Nádia Nascimento da Rosa, Dilcele Silva Moreira Dziedzic, Katherine Athayde Teixeira de Carvalho

**Affiliations:** Advanced Therapy and Cellular Biotechnology in Regenerative Medicine Department, Pelé Pequeno Príncipe Institute, Child and Adolescent Health Research and Pequeno Príncipe Faculties, Curitiba 80240-020, Paraná, Brazil; nathybarth03@gmail.com (N.B.d.O.); anairioda@gmail.com (A.C.I.); priscilaeferreira@gmail.com (P.E.F.S.); bassamfm@gmail.com (B.F.M.); nadianr@gmail.com (N.N.d.R.); dilceledz@gmail.com (D.S.M.D.)

**Keywords:** adipose-derived mesenchymal stem cells, membrane, neural precursor, polyisoprene, YAP, angiomotin

## Abstract

Adipose tissue-derived mesenchymal stem cells (ADMSCs) are promising candidates for regenerative medicine, as they have good cell yield and can differentiate into several cell lines. When induced to the neuronal differentiation, they form neurospheres composed of neural precursors (NPs) that can be an alternative in treating neurodegenerative diseases. This study aimed to characterize NPs from neurospheres obtained after seeding ADMSCs on a natural polyisoprene-based membrane. The ADMSCs were isolated from adipose tissue by enzymatic dissociation, were subjected to trilineage differentiation, and were characterized by flow cytometry for specific ADMSC surface markers. For neuronal differentiation, the cells were seeded on polystyrene flasks coated with the membrane and were characterized by immunocytochemistry and RT-PCR. The results demonstrated that the isolated cells showed characteristics of ADMSCs. At 15 to 25 days, ADMSCs seeded on the natural membrane developed neurospheres. Then, after dissociation, the cells demonstrated characteristic neuronal markers expressed on NPs: nestin, ß-III tubulin, GFAP, NeuN, and the YAP1/AMOT in the cytoplasm. In conclusion, it was demonstrated that this membrane differentiates the ADMSCs to NPs without any induction factors, and suggests that their differentiation mechanisms are related to mechanotransduction regulated by the YAP and AMOT proteins.

## 1. Introduction

Stem cells are undifferentiated cells with the ability of self-renewal and differentiation into several cell lines. They can be classified into embryonic stem cells (ESC), fetal stem cells (FSC) and adult stem cells [1]. ESCs are obtained from embryos and can differentiate spontaneously, making the control of their differentiation challenging; they also have the potential to generate teratomas, and due to their origin, there are ethical barriers that hinder their use in regenerative medicine [2,3]. FSCs can be isolated from the fetus, placenta, amniotic fluid and umbilical cord, and they are more plastic than adult stem cells [4]. Adult stem cells can be isolated from different tissues and can differentiate through supplementation with growth factors and external signals [2]. Mesenchymal stem cells (MSCs) are a population of adult stem cells that can be isolated from any vascularized tissue, have high plasticity, with the ability to differentiate into mesodermal and non-mesodermal cells; they also secrete cytokines and growth factors that provide modulation of the inflammatory response and tissue repair, contributing to the homeostasis of the organism [1,5,6,7,8]. Although there are several sources of MSCs, the amount of tissue collected is limited, and cell yield is low. For this reason, adipose-derived mesenchymal stem cells (ADMSCs) have become one of the most promising populations for regenerative medicine [9].

ADMSCs have an excellent isolation yield, a morphology similar to fibroblasts, and a linear growth rate, with overall differentiation capacity, being able to differentiate even into neuronal lineages through epigenetic factors that are regulated by the generated signaling interaction between the extracellular matrix (ECM) and the cells [10,11,12,13]. This signaling occurs through two mechanisms: biochemical signaling via phosphorylation cascades, and mechanotransduction, which can convert mechanical stimuli into a chemical response [14,15,16].

The mechanotransduction process starts at the cell membrane, the leading stimuli transmission site for the intracellular molecular processes, as the membrane is in direct contact with the substrate and ECM. Underneath the cell membrane, there are multiprotein complexes called focal adhesions that perceive and transfer mechanical cues from the extracellular environment to the cytoskeleton. The information generated by the interaction between focal adhesions and the cytoskeleton impacts chromatin-modifying proteins that modulate chromatin structure rearrangements, increasing DNA accessibility [13,17,18]. One signaling pathway that plays an essential role in mechanotransduction and epigenetic changes correlated to chromatin remodeling is the Hippo pathway, which stimulates tissue growth and homeostasis by controlling the proliferation and fate of differentiation [16]. These processes are regulated by Yes-associated protein (YAP) and transcriptional activator with PDZ binding motif (TAZ) transducers that play a crucial role in organ growth, stem cell self-renewal, and cell differentiation mediated by ECM interactions [16,19,20].

Several biomaterials are currently being used as artificial ECM, and in vitro, these matrices play a role in regulating stem cell lineage differentiation; therefore being a promising approach for regenerative therapies [21,22]. Specifically, cells grown in artificial matrices with elasticity comparable to the human brain can efficiently differentiate into neuronal lineages without the addition of neurogenic growth factors. This differentiation mechanism is related to YAP and angiomotin (AMOT) that can mediate substrate stiffness responses in MSCs during differentiation into neuronal lineages [23,24,25,26,27]. 

Therefore, this study aimed to characterize neural precursors (NPs) derived from ADMSCs cultivated on a natural biopolymer matrix obtained from the rubber extracted of the Hevea brasiliensis. This natural matrix allowed cells to form neurospheres that are composed of NPs. The neurospheres’ development can be explained by the mechanotransduction process regulated by YAP and AMOT proteins, which are translocated out of the nucleus into the cytoplasm on cells cultivated on the natural functional biopolymer matrix (NFBX) membrane. The NPs were positive for neuronal markers such as nestin, ß-III tubulin, NeuN, GFAP, and MAPT. These human NPs derived from ADMSCs could be a potential for preclinical studies of neurodegenerative diseases as cell therapy.

## 2. Materials and Methods

The Human Ethics Committee of Pequeno Príncipe Faculties approved this study, numbered: 3,049,033 on 30 November 2018. Adipose tissue samples were taken from healthy patients that underwent plastic surgeries and over 18 years old who signed the informed consent form.

### 2.1. Isolation and Culture of ADMSCs

ADMSCs were isolated as previously described [10,28,29]. Twenty samples were used; each sample was washed extensively with phosphate-buffered saline (PBS) containing 100 UI/mL penicillin and 0.1 mg/mL streptomycin, and were enzymatically digested with 0.075% type I collagenase at 37 °C, for 30 min under agitation. After incubation, collagenase type I activity was inactivated by adding an equal volume of standard culture medium composed of Dulbecco/F12 modified Eagle (DMEM/F12), 10% fetal bovine serum (FBS), 100 UI/mL penicillin and 0.1 mg/mL streptomycin. The samples were centrifuged at 600 g for 10 min, and filtered through a 100 µM mesh. Cells were plated (10^5^ cells/cm^2^) in 75 cm^2^ culture flasks with standard culture medium and incubated at 37 °C and 5% CO^2^ until they reached 80% confluence. 

When the adherent cells reached confluence, they were detached Trypsin-EDTA (0.25%) (Sigma, St. Louis, MO, USA) for 5 min. After incubation, the released cells were collected and replated for subculturing in culture flasks with standard medium. This process was repeated until the cells reached passage 4 (P4), for further analysis.

### 2.2. Characterization of ADMSCs

Cells were analyzed by flow cytometry for the expression of specific surface markers, and were submitted to the trilineage differentiation after reaching 80% confluence. After, differentiation cells were fixed with 4% paraformaldehyde (Sigma-Aldrich^®^, St. Louis, MO, USA) for 20 min for cytochemical staining procedure.

#### 2.2.1. Flow Cytometry

After trypsinization, cells were resuspended in 1 mL PBS with 5% human albumin (5% PBS/HA). The cell suspension was distributed in cytometry tubes, and the conjugated antibodies were added (Table 1), the tubes were vortexed and incubated in the dark for 15 min. After incubation, 400 µL of 5% PBS/HA was added, the tubes were vortexed again, and the supernatant was discarded. The cells were resuspended with 100 µL of 5% PBS/HA, and 5 µL of 7-AAD (7-aminoactinomycin D), used for cell viability, was added to the specific tubes that were incubated for 5 min. After incubation, 400 µL of 5% PBS/HA were added to each tube on the flow cytometer (FACS Canto II; Becton Dickinson, Franklin Lakes, NJ, USA), 10,000 cells were analyzed, and data analysis was performed using the Infinicyt™ software: Flow Cytometry Software 1.6.0 (Cytognos S.L., Santa Marta de Tormes, Spain) [28,30]. The gating strategy was carried out excluding non-viable cells (positive for the 7-AAD marker) and comparing each marker with isotypic control.

#### 2.2.2. Trilineage Tests

##### Adipogenic Differentiation

The cells were plated at a concentration of 5 × 10^3^ cells/cm^2^ and treated with standard culture medium supplemented with 0.5 μM dexamethasone (Sigma-Aldrich^®^, St. Louis, MO, USA), 0.5 mM isobutyl-methylxanthine (Sigma-Aldrich^®^, St. Louis, MO, USA), and 50 μM indomethacin (Sigma-Aldrich^®^, St. Louis, MO, USA). The cultivation with differentiation medium was maintained for 14 days and the medium was changed twice a week. The accumulation of lipid vesicles was detected by Oil Red O staining (Sigma-Aldrich^®^, St. Louis, MO, USA) [28,29].

##### Osteogenic Differentiation

The cells were plated at a density of 5 × 10^3^/cm^2^ and treated with standard culture medium supplemented with 1nM dexamethasone (Sigma-Aldrich^®^, St. Louis, MO, USA), 2 mM β-glycerol-phosphate (Sigma-Aldrich^®^, St. Louis, MO, USA), and 50 µM ascorbate-2-phosphate (Sigma-Aldrich^®^, St. Louis, MO, USA). The cells were cultured in this medium for 35 days. Mineralization was evaluated by staining the cells with 40 mM Alizarin Red (Sigma-Aldrich^®^, St. Louis, MO, USA) [28,29].

##### Chondrogenic Differentiation

The cells were resuspended at a concentration of 4 × 10^4^ cells/cm^2^, then 5 µL of the cell suspension was transferred to the center of the well of a 24-well plate and kept for two hours at 37 °C. After incubation, the differentiation medium was added according to the manufacturer’s specifications (StemPro^®^ Chondrogenesis Differentiation Kit—GIBCOTM Life Technologies, Carlsbad, CA, USA). This medium was changed twice a week for a period of 14 days, and the production of proteoglycans was stained with the Alcian Blue in acidic pH [28,31].

### 2.3. Preparation of the Polyisoprene-Based Membrane

Polyisoprene (C5H8) is the primary chemical constituent of natural rubber extracted from Hevea brasiliensis. For preparing the polyisoprene-based membrane, named as natural functional biopolymer matrix *(NFBX*) and previously described [32], the natural latex (COLITEX^®^, São Paulo, Brazil) was used in 1:2 dilution (*v*/*v*), in aqueous solution, with pure water. Then, for sterilization, this solution was exposed to ultraviolet light of Laminar Flux overnight. Thereafter, the multiwell culture plates were coated with the *NFBX*, in the proportion of 0.5 mL/cm^2^, on the polystyrene surface of the plate where cell culture occurs. After 12 h at room temperature, the culture plates were ready for cell seedings.

### 2.4. Production of NPs

To produce NPs, the ADMSCs were seeded on culture plates coated with NFBX membrane. The NFBX membrane presents an elastic surface, an inherent characteristic known of polyisoprene materials. The plating density was 2 × 10^2^ cells/mL, cultivated with the standard culture medium, and incubated at 37 °C and 5% CO_2_. After the development of neurospheres, they were transferred to polystyrene plates, without membrane, with tweezers, and then the neural precursors migrated from the neurospheres onto the polystyrene substrate. Trypsin-EDTA (0.25%) (Sigma-Aldrich^®^, St. Louis, MO, USA) was used for NPs detachment, and these cells were centrifuged at 400× *g* for 10 min at 18 °C to obtain the NPs.

### 2.5. Characterization of NPs

Both NPs and ADMSCs were characterized by immunocytochemistry and RT-PCR. The “ReNcell™ CX Human Neural Progenitor Cell Line” (Millipore Cat. No. SCC007) was used as a positive control. All analyses were performed in triplicate.

#### 2.5.1. Immunocytochemistry

The cells were characterized for the expression of the proteins listed in Table 2. 

After the neurospheres dissociation, neurosphere-derived NPs were washed with PBS and fixed with 4% paraformaldehyde for 20 min. The cells were permeabilized with 0.1% Triton X-100 (Sigma-Aldrich^®^, St. Louis, MO, USA) diluted in PBS with 1% human albumin (1% PBS/HA) for 30 min. Then, the wells were washed with PBS, the primary antibodies diluted in 1% PBS/HA were added, and the cells were incubated overnight at 4 °C. After incubation, the solutions with the primary antibodies were discarded, the cells were washed with PBS, and were incubated for 1 h at room temperature with the secondary antibody diluted in 1% PBS/HA, in the absence of light. Then, the cells were washed and 1 µg/mL of Hoechst 33258 (Invitrogen^®^M, Carlsbad, CA, USA) diluted in PBS was used to identify the nucleus [33]. The images were acquired by a high-throughput fluorescence microscopy (In Cell Analyzer 2000, GE).

#### 2.5.2. Qualitative Reverse Transcription–Polymerase Chain Reaction (RT-PCR) 

The neurospheres were grown in 6-well plates with a standard culture medium changed twice a week. When the NPs reached confluence, they were submitted to the RNA extraction protocol that was carried out following the instructions of the manufacturer of the kit “PureLink ™ RNA Mini Kit” (Invitrogen^®^M, Carlsbad, CA, USA). The complementary strand of DNA (cDNA) was produced following the instructions of the “High-Capacity cDNA Reverse Transcription Kit” kit (Invitrogen^®^M, Carlsbad, CA, USA). The RT-PCR was performed using a 20 µL system, containing the cDNA pattern and Master Mix Promega^®^. The primer’s sequences (forward and reverse), molecular weight of the amplified material, and annealing temperature are described in Table 3.

## 3. Results

### 3.1. Characterization of ADMSCs

Flow cytometry results show that the isolated cells were positive for ADMSCs characteristic surface markers (CD13, CD73, CD90, CD105), weakly positive for HLA-ABC, and were negative for HLA-DR and CD45. Less than 20% of the cells analyzed were positive for CD34 (Table S1). The histograms of sample A2 are shown in the Supplementary Material (Figure S1a,b).

#### Trilineage Test

**Figure 1:**
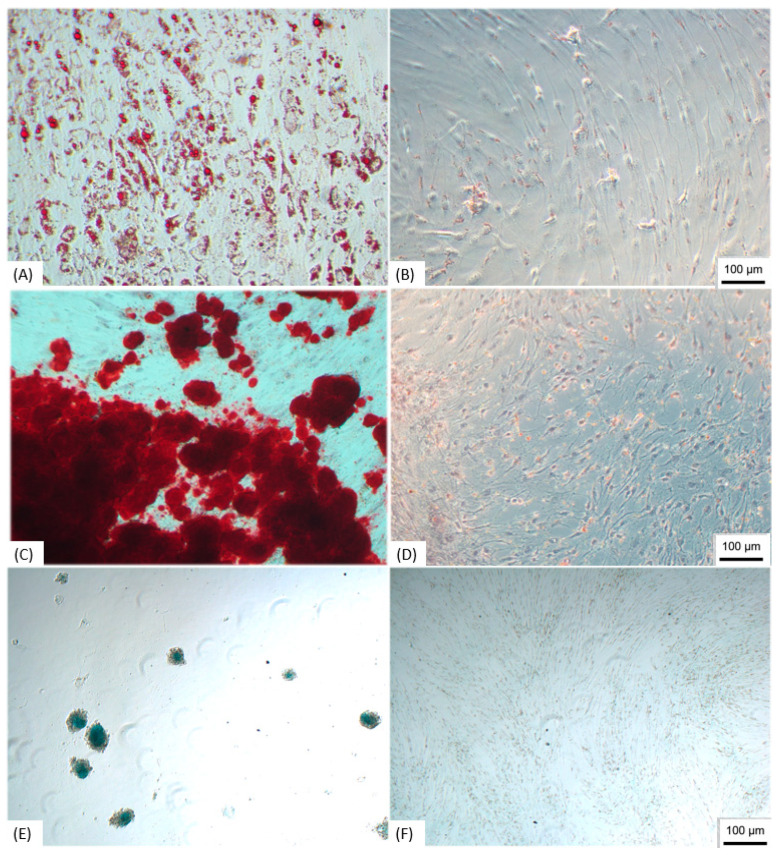
Trilineage differentiation. (**A**) ADMSCs submitted to adipogenic differentiation, staining the lipid vacuoles with Oil Red O; (**B**) Control of undifferentiated ADMSCs; (**C**) ADMSCs submitted to osteogenic differentiation showing mineralization stained with Alizarin Red; (**D**) Undifferentiated control; (**E**) ADMSCs differentiated in chondroblasts with the production of proteoglycans stained with Alcian Blue; (**F**) Undifferentiated control. Note: Image obtained by inverted optical microscopy, 100×—Axio Vert. A1, Zeiss, Germany). Scale bar, 100 µm. ADMSCs, adipose-derived mesenchymal stem cells.

ADMSCs were submitted to the trilineage differentiation, and the samples were able to differentiate in the three lineages; the adipocytes showed lipid vacuoles stained with Oil Red O (Figure 1A), in the osteoblasts it was possible to observe the mineralization through the staining with Alizarin Red (Figure 1C), and the differentiation in chondroblasts was proven by staining proteoglycans with Alcian Blue (Figure 1E), demonstrating the multipotent characteristic of isolated cells. 

ADMSCs cultured with standard medium without differentiation stimulation were used as the control, and did not show specific staining (Figure 1B,D,F).

### 3.2. Production of NPs 

**Figure 2:**
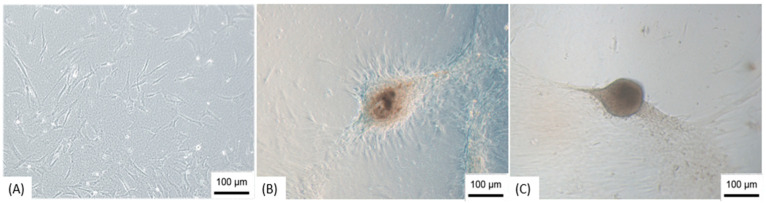
Development of neurospheres. (**A**) Cells three days after cultivation on NFBX membrane; (**B**) Neurosphere in formation; (**C**) Neurosphere ready to be dissociated. Note: Images obtained by inverted optical microscopy, 100×—Axio Vert. A1, Zeiss, Germany). Scale bars, 100 µm.

**Figure 3:**
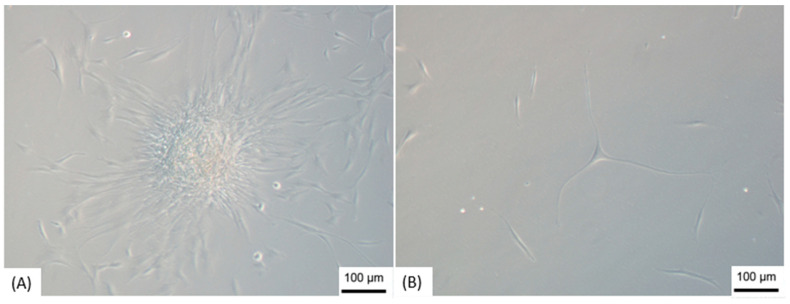
Dissociation of neurospheres. (**A**) Cells dissociating from the neurosphere; (**B**) Cells after dissociation. Note: Images obtained by inverted optical microscopy, 100×—Axio Vert. A1, Zeiss, Germany). Scale bars, 100 µm.

The differentiation of ADMSCs in NPs was through the formation of neurospheres that took place after 15–25 days of cultivation on the NFBX membrane with the standard culture medium, without the addition of neurogenic supplements (Figure 2). After the formation of the spheres, they were removed from the NFBX membrane, and transferred to plates without the membrane for NPs dissociation (Figure 3), and their confluence was reached after an average time of 15 days.

As a control, ADMSCs were grown with the same plating density on polystyrene plates without the NBFX membrane; these cells were not able to form neurospheres, suggesting that it was the fact of seeding on the NFBX membrane that allowed the development of neurospheres.

### 3.3. Immunocytochemistry

**Figure 4:**
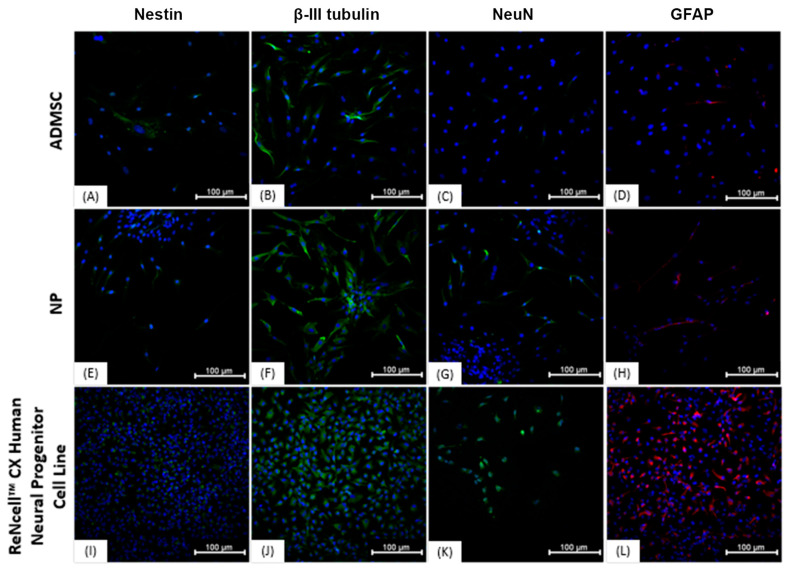
Immunocytochemistry of the ADMSCs, NPs and ReNcell™ CX. (**A**) ADMSCs labeled with anti-nestin antibody (FITC—green) and Hoechst (blue); (**B**) ADMSCs labeled with anti-𝛽-III tubulin antibody (FITC—green) and Hoechst (blue); (**C**) ADMSCs labeled with anti-NeuN antibody (FITC—green), and Hoechst (blue); (**D**) ADMSCs labeled with anti-GFAP antibody (Cy5—red), and Hoechst (blue); (**E**) NPs labeled with anti-nestin antibody (FITC—green) and Hoechst (blue); (**F**) NPs labeled with anti-𝛽-III tubulin antibody (FITC—green) and Hoechst (blue); (**G**) NPs labeled with anti-NeuN antibody (FITC—green) and Hoechst (blue); (**H**) PNs labeled with anti-GFAP antibody (Cy5—red) and Hoechst (blue); (**I**) ReNcell™ CX labeled with anti-nestin antibody (FITC—green) and Hoechst (blue); (**J**) ReNcell™ CX labeled with anti-𝛽-III tubulin antibody (FITC—green) and Hoechst (blue); (**K**) ReNcell™ CX labeled with anti-NeuN antibody (FITC—green) and Hoechst (blue); (**L**) ReNcell™ CX labeled with anti-GFAP antibody (Cy5—red) and Hoechst (blue); Images obtained with IN Cell Analyzer 2000, GE (20×). Scale bar, 100 µm. ADMSCs, adipose-derived mesenchymal stem cells; NPs, neural precursors.

**Figure 5:**
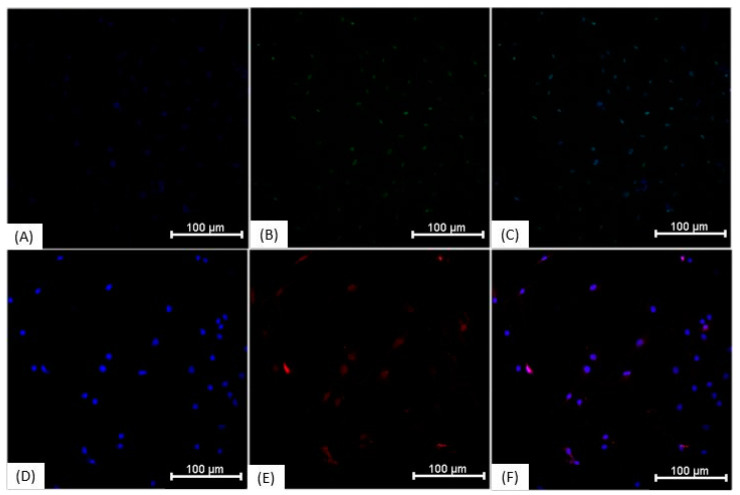
Immunocytochemistry of ADMSCs on polystyrene culture plate. (**A**) ADMSCs grown on polystyrene culture plate marking the nucleus with Hoechst (blue); (**B**) Anti-AMOT antibody with nuclear localization (FITC—green); (**C**) Overlapping images A and B; (**D**) ADMSCs grown on polystyrene culture plate marking the nucleus with Hoechst (blue); (**E**) Anti-YAP1 antibody with nuclear localization (Cy5—red); (**F**) Overlapping images D and E. The images were taken with In Cell Analyzer 2000, GE (20×). Scale bars, 100 µm. ADMSCs, adipose-derived mesenchymal stem cells.

**Figure 6:**
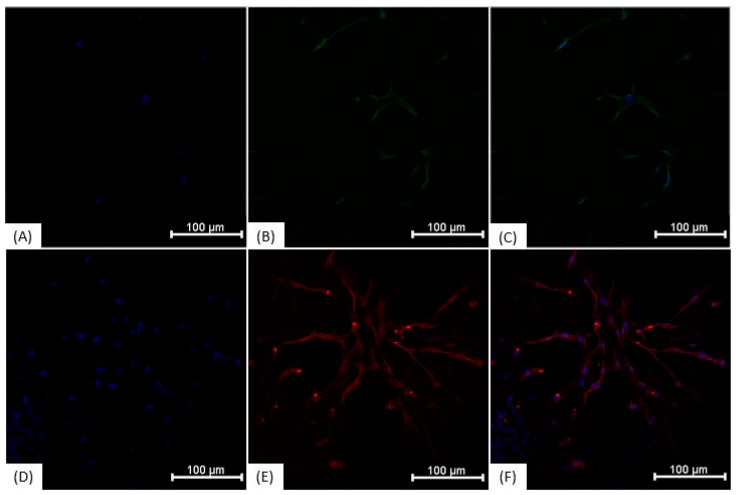
Immunocytochemistry of neurosphere-derived NPs cultivated on NFBX membrane and dissociated on polystyrene plates. (**A**) NPs cultured on NFBX membrane marking the nucleus with Hoechst (blue); (**B**) Anti-AMOT antibody with cytoplasmic labeling (FITC—green); (**C**) Overlapping images A and B; (**D**) Hoechst (blue) marking the nucleus of the cells; (**E**) Anti-YAP1 antibody with cytoplasmic labeling (Cy5—red); (**F**) Overlapping images D and E images. The images were taken with In Cell Analyzer 2000, GE (20×). Scale bars, 100 µm. NFBX, natural functional biopolymer matrix; NPs, neural precursors.

Immunocytochemical characterization was performed on NPs and on undifferentiated ADMSCs. The results demonstrate that the cells show similar protein expression (Figure 4), being positive for Nestin, ß-III tubulin, NeuN, and GFAP. The “ReNcell ^TM^ CX Human Neural Progenitor Cell Line” used as a positive control also expressed all analyzed proteins. Regarding the expression of YAP1 and AMOT proteins, in ADMSCs grown on polystyrene plates, the proteins showed nuclear localization (Figure 5), whereas neurosphere-derived NPs that were cultivated on the NFBX membrane and dissociated on polystyrene plates showed cytoplasmic protein localization (Figure 6).

### 3.4. Qualitative Reverse Transcriptio–-Polymeresae Chain Reaction (RT-PCR)

**Figure 7:**
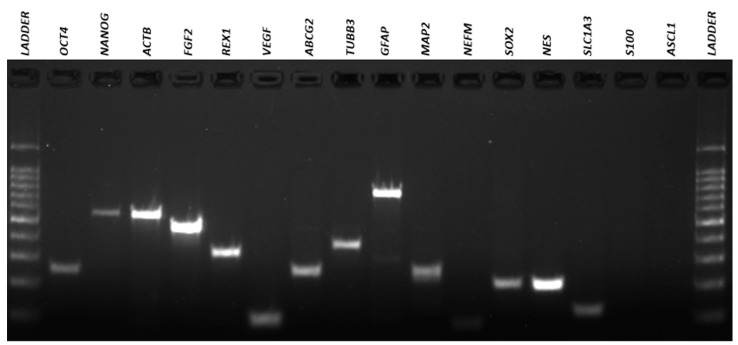
ADMSCs electrophoresis gel. RT-PCR for amplification of *OCT4* (292bp), *NANOG* (578 bp), *ACTB* (564 bp), *FGF2* (482 bp), *REX1* (344 bp), *VEGF* (107 bp), *ABCG2* (270 bp), *TUBB3* (385 bp), *GFAP* (789 bp), *MAP2* (241 bp), *NEFM* (98 bp), *SOX2* (224 bp), *NES* (220 bp), *SLC1A3* (136 bp), *S100* (261 bp) and *ASCL1* (122 bp) genes in ADMSCs. ADMSCs, adipose-derived mesenchymal stem cells.

**Figure 8:**
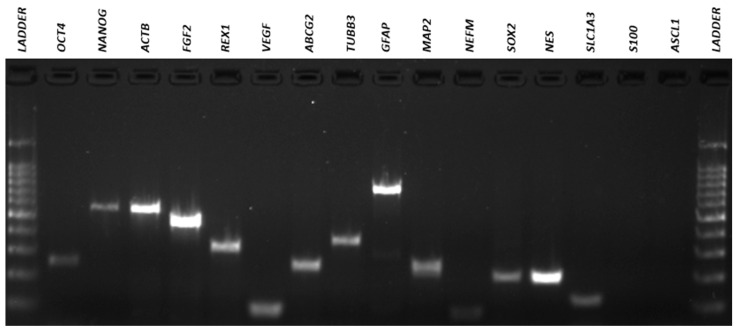
NPs electrophoresis gel. RT-PCR for amplification of *OCT4* (292 bp), *NANOG* (578 bp), *ACTB* (564 bp), *FGF2* (482 bp), *REX1* (344 bp), *VEGF* (107 bp), *ABCG2* (270 bp), *TUBB3* (385 bp), *GFAP* (789bp), *MAP2* (241bp), *NEFM* (98bp), *SOX2* (224bp), *NES* (220bp), *SLC1A3* (136bp), *S100* (261bp) and *ASCL1* (122bp) genes in NPs. NPs, neural precursors.

**Figure 9:**
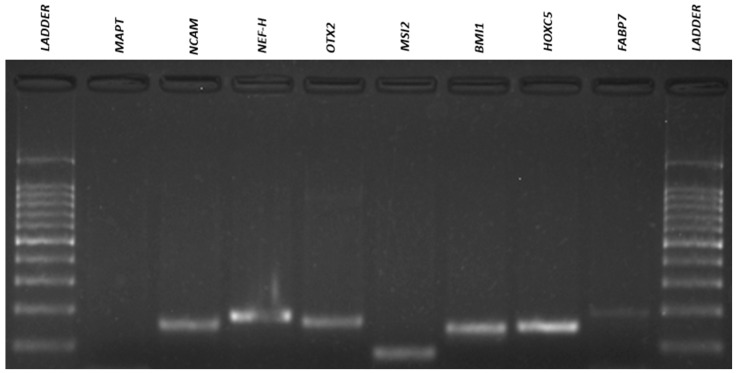
ADMSCs electrophoresis gel. RT-PCR for amplification of *MAPT* (218bp), *NCAM* (150bp), *NEF-H* (179bp), *OTX2* (163bp), *MSI2* (84bp), *BMI1* (148bp), *HOXC5* (149bp) and *FABP7* (233bp) genes in ADMSCs. ADMSCs, adipose-derived mesenchymal stem cells.

**Figure 10:**
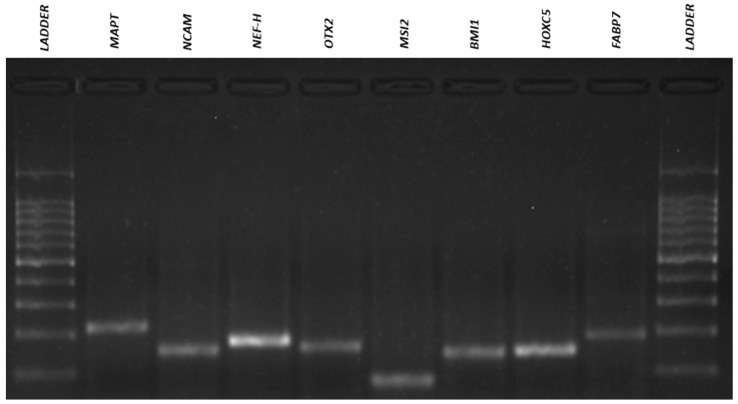
NPs electrophoresis gel. RT-PCR for amplification of *MAPT* (218bp), *NCAM* (150bp), *NEF-H* (179bp), *OTX2* (163bp), *MSI2* (84bp), *BMI1* (148bp), *HOXC5* (149bp) and *FABP7* (233bp) genes in NPs. NPs, neural precursors.

The results of the RT-PCR analysis demonstrated that neurosphere-derived NPs and undifferentiated ADMSCs have a very similar gene expression, and both cells expressed neuronal and pluripotency markers (Figure 7, Figure 8, Figure 9 and Figure 10). However, there was a difference in the expression of the MAPT gene, which was present in NPs but not in ADMSCs (Figure 9 and Figure 10). This gene is present in the axons of neurons and could suggest an increased neuronal commitment of NPs. The S100 and ASCL1 genes were not expressed by any of the cells (Figure 7 and Figure 8).

Future studies will be needed to better understand the differences in gene expression between ADMSCs and NPs, using quantitative techniques such as real-time quantitative reverse transcription PCR (qRT-PCR).

## 4. Discussion

The existence of MSCs was first recognized by Friedenstein et al. (1970), in bone marrow, and, initially, these cells were called osteogenic stem cells, due to their ability to differentiate into osteocytes. However, further studies have shown that the differentiation of these cells extends far beyond osteocytes [34,35]. Since then, many studies have focused on MSCs, precisely because of their wide capacity for differentiation, modulation of the inflammatory response, and their availability in various tissues.

The adipose tissue is considered a good source of MSCs and, like bone marrow, is derived from the embryonic mesoderm and has a heterogeneous population of cells, including ADMSCs [36]. According to the International Society for Cell Therapy, MSCs must be adherent to the rigid cell culture polystyrene plastic, capable of differentiating into adipocytes, osteoblasts, and chondroblasts, and must have characteristic cell surface markers. The minimum criteria established that more than 80% of the MSC population should be positive for CD13, CD73, CD90, and CD105, and negative (<2%) for hematopoietic markers such as CD45. CD34, on the other hand, is considered an unstable marker that can be positive in at least 20% of cells [37].

In the present study, the analysis by flow cytometry showed that the isolated cells presented the pattern of surface marker expression established in the literature and, in addition, were able to differentiate into adipocytes, osteoblasts, and chondroblasts. However, the differentiation capacity of ADMSCs does not extend only to mesodermal lineages and some studies have already shown that these cells can differentiate into neuronal lineages through epigenetic factors that can be regulated by biochemical signaling or by the mechanotransduction process. These factors have been extensively studied to understand how the physical properties of the substrate and deposited ECM can affect cell behavior [11,13,23].

Stem cell behavior is influenced by mechanical and chemical properties of ECM that have an impact on fundamental processes such as propagation, growth, proliferation, migration, differentiation, and organoid formation. The choice of the ECM type is extremely important and directly modulates the mechanotransduction of MSCs [38,39,40,41].

The cell membrane is extremely important for the mechanotransduction process, as the main force transmission site for the cell and in direct contact with the ECM. When the interaction between the cell membrane and the ECM occurs, cells develop multiprotein complexes called focal adhesions, which are essential in cell–ECM interactions. The activity of focal adhesions consists of perceiving and transferring mechanical stimuli from the extracellular environment to the cytoskeleton, which is a dynamic structure composed of filamentous and reticular proteins, responsible for providing mechanical support and controlling cell motility and shape [42,43,44].

The cytoskeleton contractility is ensured by the sliding of F-actin into myosin II, and these two proteins are held together by crosslinking proteins, called stress fibers. When a force is applied to focal adhesions, the stress fibers propagate this force from the extracellular matrix to the cell and vice versa. The generated information has an impact on proteins located in the membrane or cytoplasm, inducing their structural modification and subsequent displacement to the nucleus [17,45,46]. 

**Figure 11:**
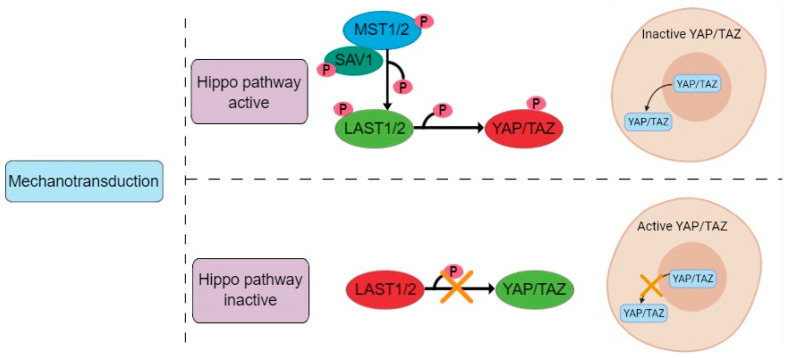
Hippo pathway regulation of YAP/TAZ. The interaction between MST1/2 and SAV1 leads to phosphorylation of LAST1/2, which are activated and phosphorylate YAP, causing its translocation outside the nucleus and cytoplasmic retention.

In this study it was proposed that cultivation on the elastic NFBX membrane surface triggered the mechanotransduction process in ADMSCs, leading to activation of the Hippo pathway, where the interaction between MST1/2 and SAV1 leads to phosphorylation of LAST1/2, which are activated and phosphorylate to YAP, causing its translocation outside the nucleus and cytoplasmic retention (Figure 11). The opposite occurs in cells grown on polystyrene plates; in this case, YAP is dephosphorylated and transported to the nucleus, where it will form a complex with DNA-binding transcription factors contributing to the control of gene expression [18,19,20,47]. The accumulation of YAP in the cytoplasm and its degradation are regulated through phosphorylation by LAST1/2 kinases, and AMOT can act as a scaffold protein to promote YAP phosphorylation [24,26]. AMOT is considered essential for the control of YAP localization and can inhibit or promote its activity, mainly in the nucleus, being responsible for mediating responses regarding the actin cytoskeleton state and the sub-cellular location of YAP during neuronal differentiation [25].

**Figure 12:**
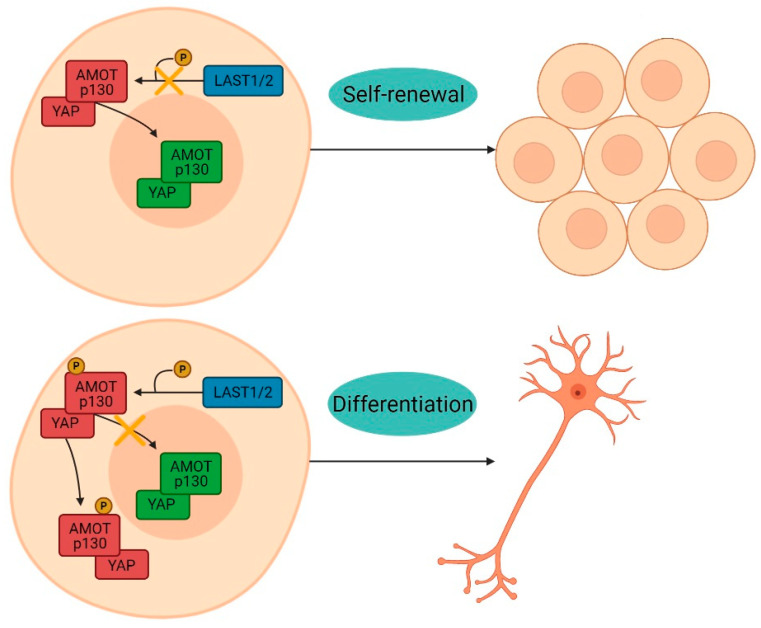
YAP/AMOT interaction. When phosphate-binding does not occur, both AMOT and YAP move to the nucleus, where YAP promotes cell proliferation. However, when phosphate is attached at the serine 176 position, AMOT predominantly interacts with YAP on the cell surface to stimulate neuronal differentiation.

AMOT was originally identified as an angiostatin-binding protein involved in the regulation of endothelial cell polarization, migration, proliferation, and angiogenesis. It is expressed as two isoforms, generated by alternative splicing: AMOT-p130, which is associated with actin and therefore contains the binding sites for F-actin and YAP, and AMOT-p80, a shorter form of the increased protein responsible for cell migration [48]. When phosphate is attached at the position known as serine 176, AMOT predominantly interacts with YAP on the cell surface to stimulate neuronal differentiation [19,25]. However, when phosphate-binding does not occur, both AMOT and YAP move to the nucleus, where YAP promotes cell proliferation (Figure 12) [19,25,26]. In the present study, the culture on the NFBX membrane induced the cytoplasmic localization of YAP and AMOT proteins, indicating neuronal differentiation. In the polystyrene plates, the proteins maintained their nuclear location, promoting cell proliferation.

After the formation of neurospheres, they were removed from the NFBX membrane and seeded again in polystyrene plates for the dissociation of the NPs for four days or up to two weeks, and, through immunocytochemical analysis, it was possible to observe that even after transferring the cells to plates without the NFBX membrane, the behavior of the proteins YAP and AMOT was the same, remaining in the cytoplasm. Therefore, further studies will be needed to understand how far the role of YAP and AMOT extends and whether long-term cultivation on polystyrene plates could cause the opposite effect, leading the cells to return to their previous state.

Cells grown on the NFBX membrane formed neurospheres that are crucial for the efficiency of neuronal differentiation [49]. Most protocols for the production of neurospheres use high-cost supplements or gene transfection; however, this study used only cultivation on the NFBX membrane with standard culture medium, without any supplementation or gene transfection. This protocol is considered promising because of its low cost and because it provides a less interfering environment. However, the time for neurosphere formation is longer (15–25 days) than that reported in studies which used supplementation with neurogenic factors (7–9 days) [50,51,52].

Immunocytochemical analysis demonstrated that both neurosphere-derived NPs and undifferentiated ADMSCs expressed characteristic neuronal proteins. ADMSCs express early and late neuronal markers, and about 1–5% of cells are positive for Nestin, but this expression decreases with the number of passages. GFAP can be positive in 10–75% of cells depending on the number of passages, while NeuN is positive in up to 70% of cells and 𝛽III-tubulin is present in about 90% of cells, regardless of the number of passages [53]. Although both cell types expressed the neuronal markers, the NPs showed a better marking for the NeuN and GFAP proteins.

Characterization by RT-PCR revealed that NPs and ADMSCs have very similar gene expression and most of the genes analyzed were expressed by both cell types, with the exception of the MAPT gene which was expressed by NPs, but not by ADMSCs. The MAPT gene encodes the microtubule-associated tau protein that is primarily expressed in the brain and is present in the axons of neurons and stabilizes the microtubule bundles, playing a central role in their dynamics, regulating their assembly, behavior, and spatial organization [54,55]. MAPT presence in NPs could suggest neuronal commitment. Recent study also demonstrated that NPs cultivated in the NFBX membrane were able to differentiate into cholinergic-like neurons, substantiating that these NPs can differentiate into more mature cells [32]. 

When comparing NPs derived from ADMSC with “ReNcell™ CX Human Neural Progenitor Cell Line”, we can observe that the *ABCG2* gene is present in NPs, but not in the lineage cells used as a positive control (see Supplementary Material, Figure S2). This difference could be related to the different origin of these two cells, as the NPs are derived from ADMSCs and thus tend to maintain similar characteristics to their source. ADMSCs express neuronal marker genes at the transcription level and, after neurogenic induction, this expression increases [56]. Therefore, future studies will be needed to quantify the expression of analyzed genes, as well as proteins.

## 5. Conclusions

This study demonstrated that ADMSCs were able to differentiate into cells with neuronal phenotype characteristics through cultivation on the elastic NFBX membrane without the addition of neurogenic growth factors or gene transfection. This neuronal differentiation could be related to epigenetic factors, or more specifically, the mechanotransduction capacity of cells regulated by YAP and AMOT proteins. 

## Figures and Tables

**Table 1:** Antibodies for flow cytometry.

Antigen	Clone	Fluorochrome
CD13	SJ1D1	PE
CD34	581	PE-CY7
CD45	2D1	APC-CY7
CD73	AD2	APC
CD90	5E10	FITC
CD105	266	PE
HLA-DR	L243	FITC
HLA-ABC	G46-2.6	APC

**Table 2:** The antibodies for immunocytochemistry.

Antibody	Dilution	Code
Nestin	1:300	S1409
𝛽-III tubulin	4 µg/mL	T2200
GFAP	1:300	G3893
NeuN	1:50	SAB4300883
YAP1	20 µg/mL	WH0010413M1
AMOT	2 µg/mL	HPA067853
Anti-rabbit secondary antibody with FITC	10 μg/mL	F7512
Anti-mouse secondary antibody with Cy5	10 μg/mL	A10524

Note: The antibodies were obtained from Sigma Aldrich^®^ (St. Louis, MO, USA). FITC, Fluorescein isothiocyanate; Cy5, Cyanine-5.

**Table 3:** Primers for RT-PCR.

Gene	Forward (5′-3′)	Reverse (5′-3′)	AT (°C) ^1^	MW (bp) ^2^
*OCT4*	AGCCCTCATTTCACCAGGCC	CCCCCACAGAACTCATACGG	62	292
*NANOG*	TCCAGGATTTTAACGTTCTGCT	TTCTTGCATCTGCTGGAGGC	60	578
*ACTB*	CTGGGACGACATGGAGAAAA	AAGGAAGGCTGGAAGAGTGC	56	564
*FGF2*	TGCTGGTGATGGGAGTTGTA	CCTCCAAGTAGCAGCCAAAG	60	482
*REX1*	CTGAAGAAACGGGCAAAGAC	GAACATTCAAGGGAGCTTGC	60	344
*VEGF*	AGCCTTGTTCAGAGCGGAGAA	TAACTCAAGCTGCCTCGCCTT	60	107
*ABCG2*	GTCTAAGCAGGGACGAACAATC	GGCTCTATGATCTCTGTGGCTT	62	270
*TUBB3*	GGAGATCGTGCACATCCAGG	CAGGCAGTCGCAGTTTTCAC	62	385
*SOX2*	ACACCAATCCCATCCACACT	GCAAACTTCCTGCAAAGCTC	62	224
*MAPT*	GGCTACACCATGCACCAAGA	CCTTCTGGGATCTCCGTGTG	62	218
*NCAM*	CCTGAAGCCCGAAACAAC	TTTCCATCCTCTCCCATCT	58	150
*OTX2*	CTCTGAACCTGTCCACCC	AGCAAGTCCATACCCGAA	60	163
*NEF-H*	GACATTGCCTCCTACCAG	AAGCCAATCCGACACTCT	58	179
*GFAP*	CTCACCAAATTCCACCCGCA	ACCGCACACAGTACCTGAAG	60	769
*MAP2*	GCTAAATCGTAAGTGAGGGCTG	TGGCTCTCTGGCTCTCTAGC	60	241
*NEFM*	ACATCGAGAGCGCCACAA	GACGAGCCATTTCCCACTTTG	60	98
*SLC1A3*	GGCGGCGCTAGATAGTAAGG	TTCCTTTGTGCCCTTCCCAG	62	136
*S100*	GGAGACAAGCACAAGCTGAA	CTTGCATGACCGTCTCTGTT	58	261
*ASCL1*	CGAACTGATGCGCTGCAAA	TGACCAACTTGACGCGGTT	58	122
*HOXC5*	TCAAAGAGTCACAAATCACCC	ATCCATAGTTCCCACAAGTT	58	149
*FABP7*	GTGGTCATCAGGACTCTCAGC	CGAACAGCAACCACATCACC	62	233
*MSI2*	CCCCAAAGTTGCATTTCCTCG	TTCGCAGATAACCCGCCTAC	60	84
*BMI1*	CGCTTGGCTCGCATTC	AGCTCAGTGATCTTGATTCTCGTTG	62	148
*NES*	AACAGCGACGGAGGTCTCTA	TTCTCTTGTCCCGCAGACTT	58	220

Note: Primers obtained from Sigma Aldrich^®^. ^1^ AT (°C) = primer’s annealing temperature in degrees Celsius; ^2^ MW (bp) = molecular weight in base pairs.

## Data Availability

Not applicable.

## Supplementary Materials

The following are available online at https://www.mdpi.com/article/10.3390/membranes11090687/s1, Figure S1: Flow cytometry histograms; Figure S2: Immunocytochemistry of ReNcell™ CX Human Neural Progenitor Cell Line; Figure S3: ReNcell™ CX Human Neural Progenitor Cell Line electrophoresis gel-1; Figure S4: ReNcell™ CX Human Neural Progenitor Cell Line electrophoresis gel-2.
