# Supplementary material for: Natural Membrane Differentiates Human Adipose-Derived Mesenchymal Stem Cells to Neurospheres by Mechanotransduction Related to YAP and AMOT Proteins
Source: Membranes (Basel). 2021 Sep 5;11(9):687. doi: 10.3390/membranes11090687 (PMC8469618; doi:10.3390/membranes11090687)
Supplement: Supplementary file 1 [file membranes-11-00687-s001.zip › membranes-1348394-supplementary.pdf]

Supplementary Material

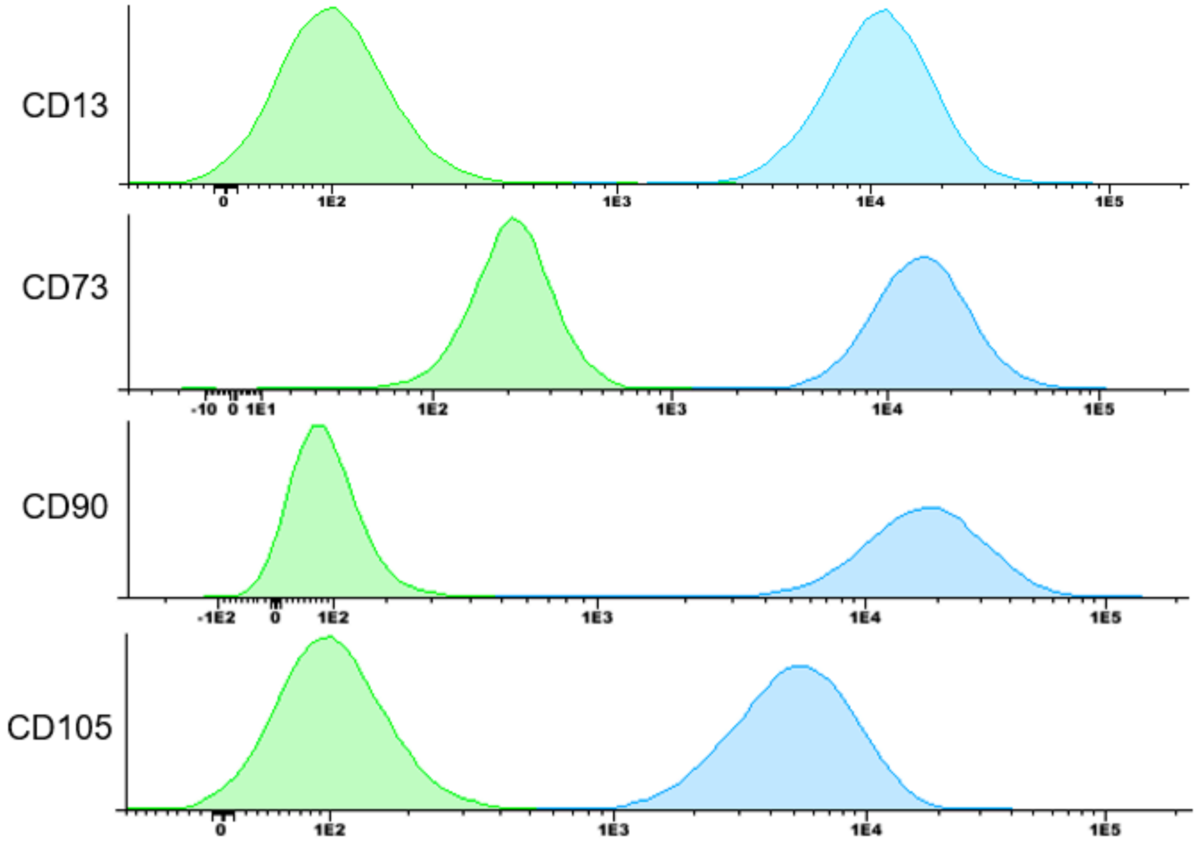

Figure S1a. Flow cytometry Histograms. Note: The isotypic control is represented by the green peak that delimits the area in which the samples are negative for the markers. The blue peak represents the cell population analyzed. The histograms show that the population is positive for CD13, CD73, CD90 and CD105.

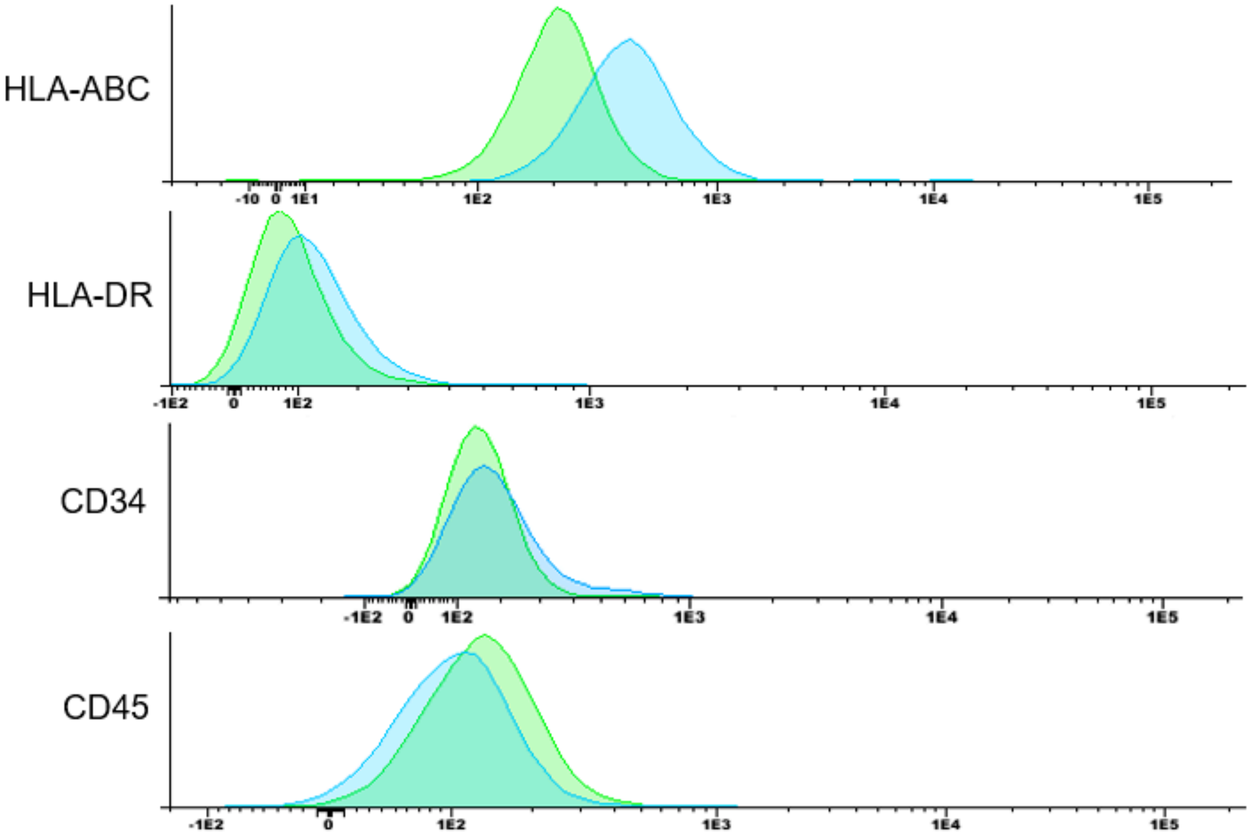

Figure S1b. Flow cytometry Histograms. Note: The isotypic control is represented by the green peak that delimits the area in which the samples are negative for the markers. The blue peak represents the cell population analyzed. The histograms show that the population is weakly positive for HLA-ABC, and negative for CD34, CD45, and HLA-DR.

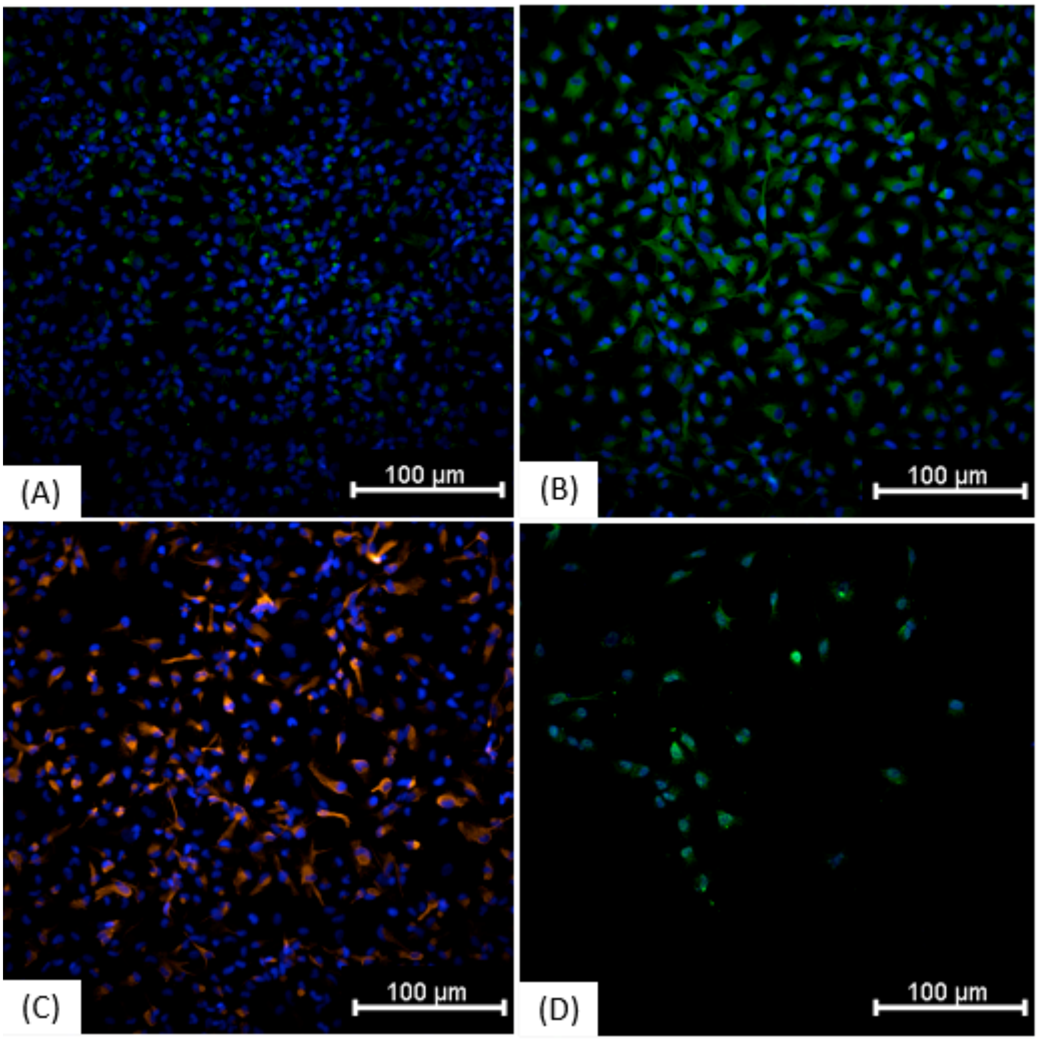

Figure S2. Immunocytochemistry of ReNcell™ CX Human Neural Progenitor Cell Line. (**A**) Cells labeled with anti-nestin antibody (FITC - green) and Hoechst (blue); (**B**) Cells labeled with anti-β-III tubulin antibody (FITC - green) and Hoechst (blue); (**C**) Cells labeled with anti- GFAP antibody (Cy5 - red) and Hoechst (blue); (**D**) Cells labeled with anti-NeuN antibody (FITC - green) and Hoechst (blue); Image obtained with In Cell Analyzer 2000, GE (20X). Scale bar, 100 µm.

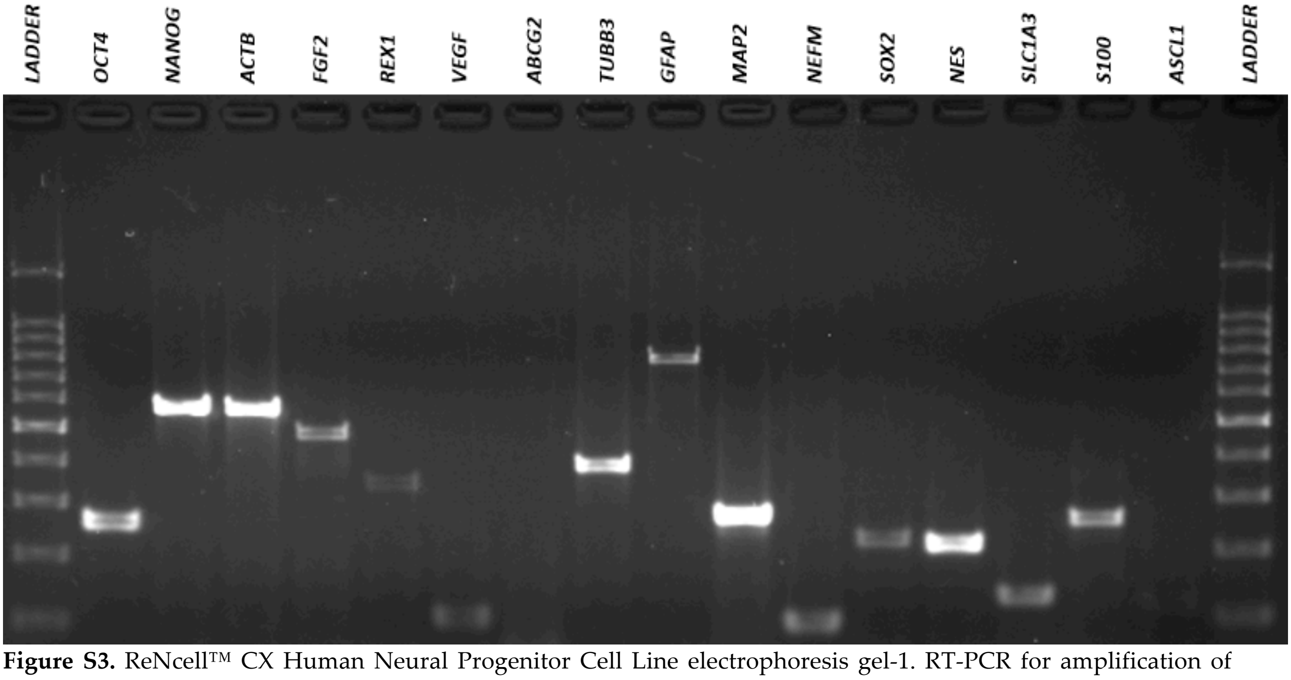

Figure S3. ReNcell™ CX Human Neural Progenitor Cell Line electrophoresis gel-1. RT-PCR for amplification of OCT4 (292bp), NANOG (578bp), ACTB (564bp), FGF2 (482bp), REX1 (344bp), VEGF (107bp), ABCG2 (270bp), TUBB3 (385bp), GFAP (789bp), MAP2 (241bp), NEFM (98bp), SOX2 (224bp), NES (220bp), SLC1A3 (136bp), S100 (261bp) and ASCL1 (122bp) genes.

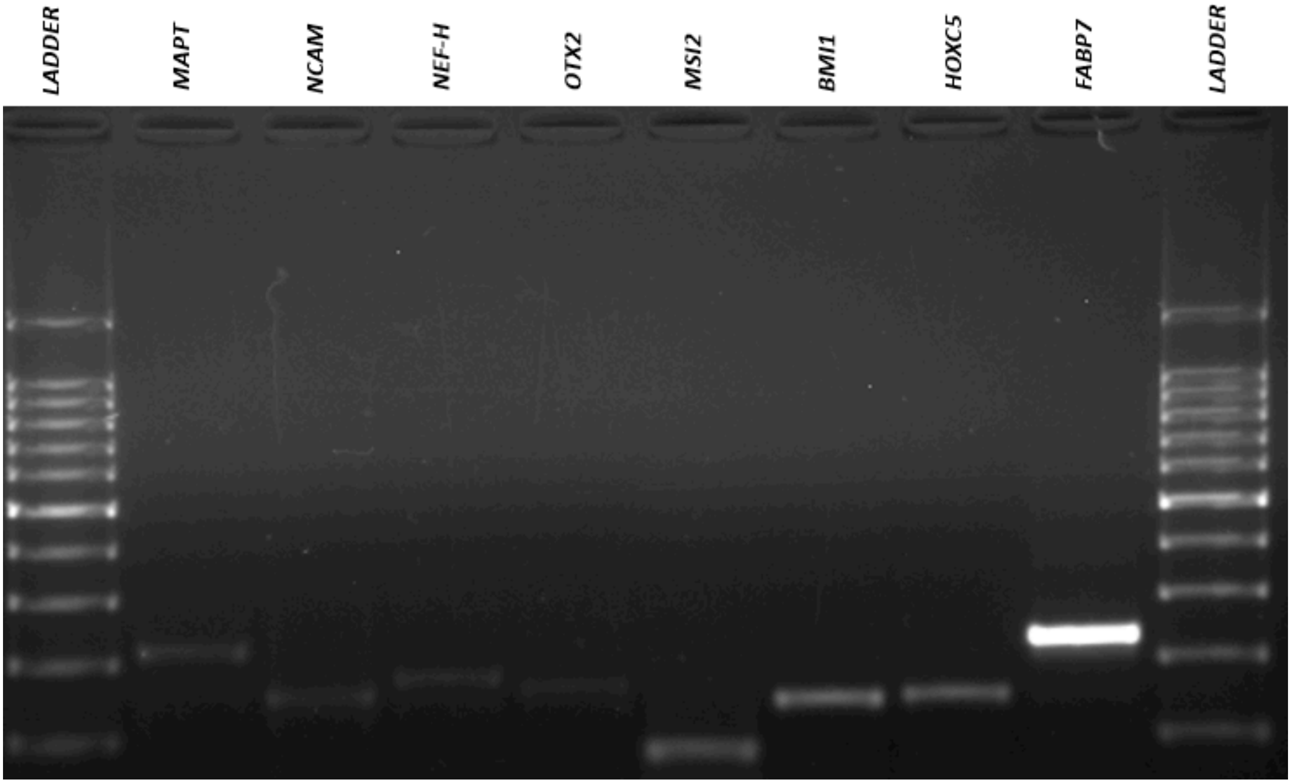

Figure S4. ReNcell™ CX Human Neural Progenitor Cell Line electrophoresis gel-2. RT-PCR for amplification of *MAPT* (218bp), *NCAM* (150bp), *NEF-H* (179bp), *OTX2* (163bp), *MSI2* (84bp), *BMI1* (148bp), *HOXC5* (149bp) and *FABP7* (233bp) genes.
